# Redox Homeostasis Alteration Is Restored through Melatonin Treatment in COVID-19 Patients: A Preliminary Study

**DOI:** 10.3390/ijms25084543

**Published:** 2024-04-21

**Authors:** María Elena Soto, Israel Pérez-Torres, Linaloe Manzano-Pech, Adrían Palacios-Chavarría, Rafael Ricardo Valdez-Vázquez, Verónica Guarner-Lans, Elizabeth Soria-Castro, Eulises Díaz-Díaz, Vicente Castrejón-Tellez

**Affiliations:** 1 Research Direction, Instituto Nacional de Cardiología Ignacio Chávez, Juan Badiano 1, Sección XVI, Tlalpan, Mexico City 14080, Mexico; mesoto50@hotmail.com; 2 Department of Cardiovascular Biomedicine, Instituto Nacional de Cardiología Ignacio Chávez, Mexico City 14080, Mexico; loe_mana@hotmail.com (L.M.-P.); elizabeth.soria@cardiologia.org.mx (E.S.-C.); 3 Critical Care Units, Temporal COVID-19 Unit, Citibanamex Center, Mexico City 11200, Mexico; a2novi@hotmail.com (A.P.-C.); rrvaldezvazquez@gmail.com (R.R.V.-V.); 4 Department of Physiology, Instituto Nacional de Cardiología Ignacio Chávez, Juan Badiano 1, Sección XVI, Tlalpan, Mexico City 14080, Mexico; veronica.guarner@cardiologia.org.mx (V.G.-L.); 5 Department of Reproductive Biology, Instituto Nacional de Ciencias Médicas y Nutrición Salvador Zubirán, Vasco de Quiroga 15, Sección XVI, Tlalpan, Mexico City 14000, Mexico; eulises.diazd@incmnsz.mx

**Keywords:** melatonin, SARS-CoV-2, oxidative stress, COVID-19, redox homeostasis, antioxidant enzymes

## Abstract

Type II pneumocytes are the target of the SARS-CoV-2 virus, which alters their redox homeostasis to increase reactive oxygen species (ROS). Melatonin (MT) has antioxidant proprieties and protects mitochondrial function. In this study, we evaluated whether treatment with MT compensated for the redox homeostasis alteration in serum from COVID-19 patients. We determined oxidative stress (OS) markers such as carbonyls, glutathione (GSH), total antioxidant capacity (TAC), thiols, nitrites (NO_2_^−^), lipid peroxidation (LPO), and thiol groups in serum. We also studied the enzymatic activities of glutathione peroxidase (GPx), glutathione-S-transferase (GST), reductase (GR), thioredoxin reductase (TrxR), extracellular superoxide dismutase (ecSOD) and peroxidases. There were significant increases in LPO and carbonyl quantities (*p* ≤ 0.03) and decreases in TAC and the quantities of NO_2_^−^, thiols, and GSH (*p* < 0.001) in COVID-19 patients. The activities of the antioxidant enzymes such as ecSOD, TrxR, GPx, GST, GR, and peroxidases were decreased (*p* ≤ 0.04) after the MT treatment. The treatment with MT favored the activity of the antioxidant enzymes that contributed to an increase in TAC and restored the lost redox homeostasis. MT also modulated glucose homeostasis, functioning as a glycolytic agent, and inhibited the Warburg effect. Thus, MT restores the redox homeostasis that is altered in COVID-19 patients and can be used as adjuvant therapy in SARS-CoV-2 infection.

## 1. Introduction

The type 2 coronavirus that causes severe acute respiratory syndrome (SARS-CoV-2), responsible for the COVID-19 pandemic, continues to wreak havoc amidst the population around the world. As of January 2024, a total of 773,449,299 cases had been reported, causing 6,991,842 deaths, despite the administration of 13.59 billon doses of vaccines according to the WHO Coronavirus (COVID-19) Dashboard. Alveolar epithelial type 2 cells are the first targets of SARS-CoV-2 infection through the attachment of the S protein of the virus with the angiotensin-converting enzyme 2 (ACE2) receptor, interacting with its trans membrane serine protease type 2 (TMPRSS2), which is a trans membrane-bound protease expressed in lung and bronchial cells [1]. The union of the virus with this receptor facilitates the fusion of the viral membrane with the cellular membrane of the type II pneumocytes, and subsequently, the virus hijacks the cellular machinery to complete the replication of new virions [2]. In this process, the host progressively triggers a cytokine storm through the innate immune system to try to counteract the infection, resulting in excessive systemic inflammation, which is accompanied by an increase in the number of radical oxygen species (ROS), lactate creatinine, hyperferretinemia, lymphopenia, and kinase dehydrogenase. The ROS overproduction induces, in turn, the activation of NFkB, which promotes an increased production of cytokines that enhance the inflammatory response. This leads to a positive feedback loop that promotes acute lung injury (ALI), which rapidly progresses to acute respiratory distress syndrome (ARDS) resulting in COVID-19 [3]. The clinical presentations of COVID-19 range from asymptomatic cases to severe pneumonia associated with ARDS and cardiogenic shock. The severe forms are more often present in the elderly population and in patients with the chronic pathologies that comprise the metabolic syndrome [4].

Despite the availability of various antiviral agents and various vaccines against SARS-CoV-2, the search for adjuvant therapies that can contribute to reducing the severity of the infection through clinical and basic studies continues, with the aim of lowering the viral load that is the source and origin of chronic inflammation, the cytokine storm, and the excessive ROS production [5]. In this sense, the use of antioxidants such as melatonin (MT) could decrease the exacerbated inflammatory response, the cytokine storm, and the oxidative stress (OS) and correct the alteration in redox homeostasis.

MT (N-acetyl-5-methoxytryptamine) is an indoleamine synthesized from the amino acid serotonin. The enzymes that participate in its synthesis are of the type arylalkylamine N-acetyl transferase, which converts serotonin to N-acetyl serotonin, which is then converted to MT by acetyl serotonin methyl transferase [6]. MT is also an endocrine molecule secreted by the pineal gland in response to darkness that is associated with the circadian cycle and secreted into the cerebrospinal fluid and blood. It has effects in all the cells and tissues [7]. MT has two receptors, MT1 and MT2, which have a high affinity and can be triggered even at low concentrations of MT [8]. MT can also join with nuclear RAR-related orphan receptors and the retinoid Z receptors [9]. However, the molecule of MT is amphiphilic, and therefore, it can also cross the cell membrane. MT is also synthesized in the mitochondria [10]. However, MT production can be suppressed by the cytokine storm associated with severe influenza and SARS-CoV-2 infection in the pineal gland and the mitochondria. This contributes to the deregulation of mitochondrial metabolism, increasing the pro-inflammatory state and unbalancing the redox homeostasis that is altered by an increase in the ROS volume [11].

MT prevents the overproduction of ROS through its pyrrole ring structure, which provides it with a high capacity to entrap O_2_^−^ and hydroxyl (OH^−^) radicals [12]. The sequestration of ROS protects cell membrane lipids, cytosol proteins, and nuclear and mitochondrial DNA [13]. MT can also chelate metal ions such as Fe^3+^ that are involved in the Fenton and Haber–Weiss reactions, which are series of autocatalytic reactions by which the ferrous ion decomposes hydrogen peroxide to OH^−^ groups, thus decreasing the formation of the OH^−^ that participates in lipid peroxidation (LPO) [14]. MT may also stimulate the antioxidant enzymatic system [15], inhibiting the enzymes that contribute to ROS production such as myeloperoxidase (MPO), lipoxygenase, and NADPH oxidases [16]. MT increases the efficiency of the transfer of electrons between the mitochondrial respiratory complexes, thereby decreasing electron leakage and super oxide formation [17]. It also stabilizes the integrity of the mitochondrial inner membrane [18]. It may increase glutathione (GSH) synthesis through the regulation of the γ-glutamyl cysteine synthetase [19].

On the other hand, OS is characterized by an increase in ROS and the depletion of the enzymatic and non-enzymatic antioxidant systems. This leads to the disturbance of redox homeostasis [20]. Therefore, the objective of this study was to evaluate and demonstrate whether treatment with MT contributes to decreasing the OS present in COVID-19 patients and whether it may correct the alteration in redox homeostasis.

## 2. Results

### 2.1. Demographic Characteristics of the COVID-19 Patients

A total of thirteen (76.47%) men and four (23.52%) women were included in the group of patients, adding to a total of seventeen subjects. The demographic characteristics are shown in Table 1. The values are expressed as medians and with minimum and maximum ranges.

Table 2 shows the values of glucose and insulin and the homeostasis model assessment index (HOMA index) in the COVID-19 patients before and after the treatment with MT. It was observed that after MT treatment, there was a significant decrease in the glucose concentration (*p* = 0.05). The values of IL-6 and high-density lipoprotein (HDL) are also presented in the same table. MT treatment decreased the IL-6 levels (*p* = 0.02), and there was a tendency to decrease in the HDL (*p* = 0.09) but without significant difference. Values for insulin in ng/mL = 1.30 and 0.10–10.10; HOMA index = 9.71 and 16.70–100.15; total cholesterol in mg/dL = 141 and 76–196; triglycerides in mg/dL = 140 and 67–325; and low-density lipoprotein (LDL) in mg/dL = 83 and 40–108 did not show significant changes after the treatment with MT and are also shown.

### 2.2. Blood Biochemical Characteristics of the Healthy Subjects

Fifteen (75%) men and five (25%) women were included in the group of healthy subjects, adding up to a total of twenty individuals. Healthy subjects had a median age of 57 with min 27 and max 87 ranges. The values of the levels of glucose, uric acid, cholesterol, HDL, LDL, triglycerides, and C-reactive protein and the atherogenic index are shown in Table 3.

### 2.3. Activities of the Enzymes That Use Glutathione

**Figure 1:**
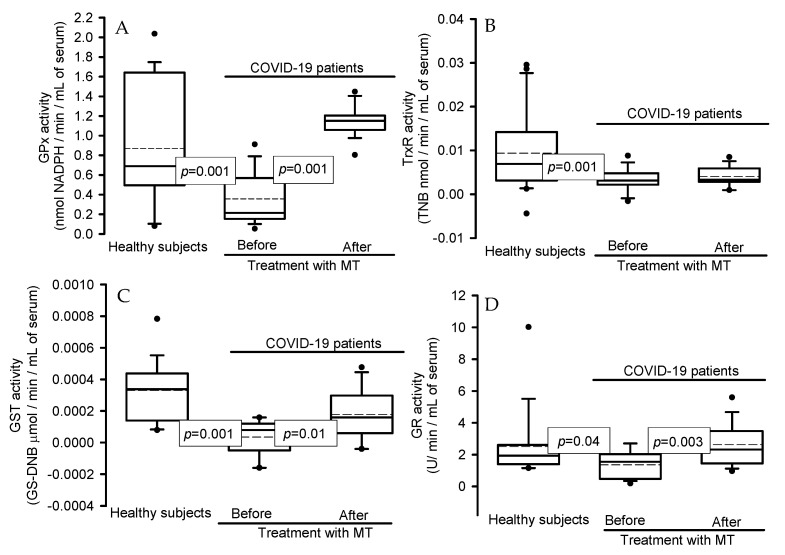
Activities of the four antioxidant enzymes that employ glutathione in COVID-19 patients before and after the treatment with melatonin. (**A**) GPx activity; the 95% confidence interval was, for healthy subjects, of 0.30 and for patients with COVID-19, before treatment with MT, of 0.13 and after treatment of 0.07. (**B**) TrxR. The 95% confidence interval was, for healthy subjects, of 4.0^−3^ and for patients with COVID-19, before treatment with MT, of 1.3^−3^ and after treatment of 1.2^−3^. (**C**) GST activity. The 95% confidence interval was, for healthy subjects, of 8.6^−5^ and for patients with COVID-19, before treatment with MT, of 5.1^−5^ and after treatment of 8.3^−5^. (**D**) GR activity. The 95% confidence interval was, for healthy subjects, of 0.98 and for patients with COVID-19, before treatment with MT, of 0.44 and after treatment of 0.67. Abbreviations: GPx = glutathione peroxidase, TrxR = thioredoxin reductase, GST = glutathione-S-transferase, and GR = glutathione reductase. The dot is the outliers and the dotted line is the mean of the values.

Figure 1 shows the activities of the enzymes that use glutathione (GSH), including the following: glutathione peroxidase (GPx) (*p* = 0.001), thioredoxin reductase (TxrR) (*p* = 0.001), glutathione-S-transferase (GST) (*p* = 0.001), and glutathione reductase (GR) (*p* = 0.04). These showed decreases in the serum of the COVID-19 patients in comparison with healthy subjects. However, the treatment with MT favored a significant increase in the activities of these enzymes: GPx (*p* = 0.001), GST (*p* = 0.01), and GR (*p* = 0.003). However, the TxrR activity only showed a tendency to increase without reaching statistical significance.

### 2.4. Oxidative Stress Markers

**Figure 2:**
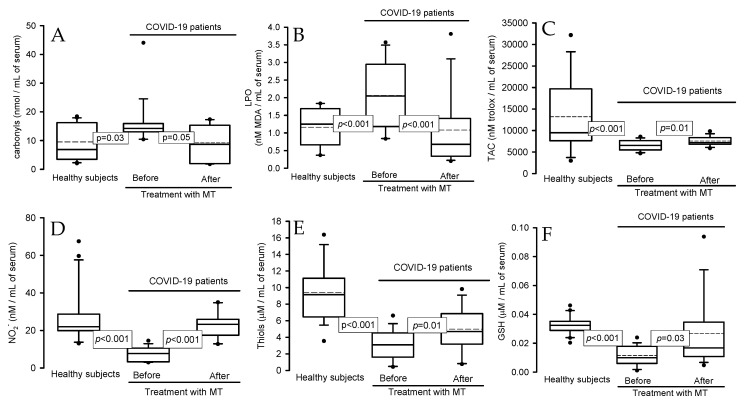
Oxidative stress markers in healthy subjects and patients with COVID-19 before and after treatment with MT. The total carbonyl concentration and LPO index increased in the serum of COVID-19 patients compared to healthy subjects, but MT treatment decreased the carbonyl concentration and LPO index in COVID-19 patients. The TAC and NO_2_^−^, thiol, and GSH concentration groups showed significant decreases in COVID-19 patients compared to healthy subjects, but after treatment with MT, these variables increased. (**A**) Carbonyls, (**B**) lipid peroxidation index, (**C**) total antioxidant capacity, (**D**) nitrites, (**E**) thiol groups and (**F**) reduced glutathione. Abbreviations: LPO = lipid peroxidation, TAC = total antioxidant capacity, NO_2_^−^ = nitrites, and GSH = reduced glutathione. The dot is the outliers and the dotted line is the mean of the values.

The total carbonyl concentration and the lipid peroxidation (LPO) index were increased in the serum of the COVID-19 patients in comparison with healthy subjects (*p* = 0.03, *p* < 0.001) (Figure 2A and Figure 2B, respectively), but the treatment with MT decreased the concentration of carbonyls and LPO index (*p* = 0.05 and *p* < 0.001). The total antioxidant capacity (TAC), NO_2_^−^, levels of thiol groups, and GSH concentration showed significant decreases in the COVID-19 patients in comparison with healthy subjects (*p* < 0.001), but these variables were increased after the treatment with MT (*p* = 0.01, *p* < 0.001, and *p* = 0.0, respectively; Figure 2C–F).

### 2.5. Activities of Enzymes: Peroxidases and ecSOD in Native Gels

**Figure 3:**
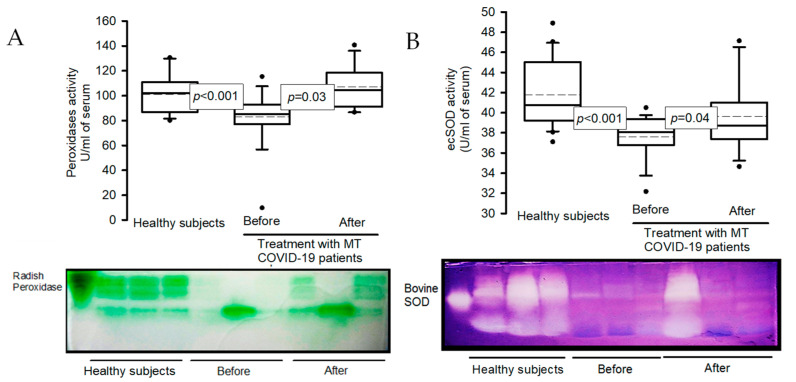
Activity of the peroxidases (**A**) and ecSOD (**B**) in healthy subjects and COVID-19 patients before and after treatment with MT. The activities of both enzymes were evaluated on native polyacrylamide gels at 10%. In the first line of both gels, the activity of the commercial enzyme was observed (radish peroxidase and bovine SOD-2, respectively): see the graphic shown in blot boxes and whiskers in percentiles 75 and 25, median, midline, and outliers. Abbreviations: ecSOD = extracellular superoxide dismutase, MT = melatonin. The dot is the outliers and the dotted line is the mean of the values.

Figure 3A shows that the activity of peroxidases in the COVID-19 patients presented a significant decrease in comparison with healthy subjects (*p* < 0.001), but the treatment with MT increased this (*p* = 0.03). The same tendency was present in the extracellular super oxide dismutase (ecSOD) activity in the COVID-19 patients in comparison with healthy subjects (*p* < 0.001) after the treatment with MT (*p* = 0.04, Figure 3B).

### 2.6. Electron Microscopy

**Figure 4:**
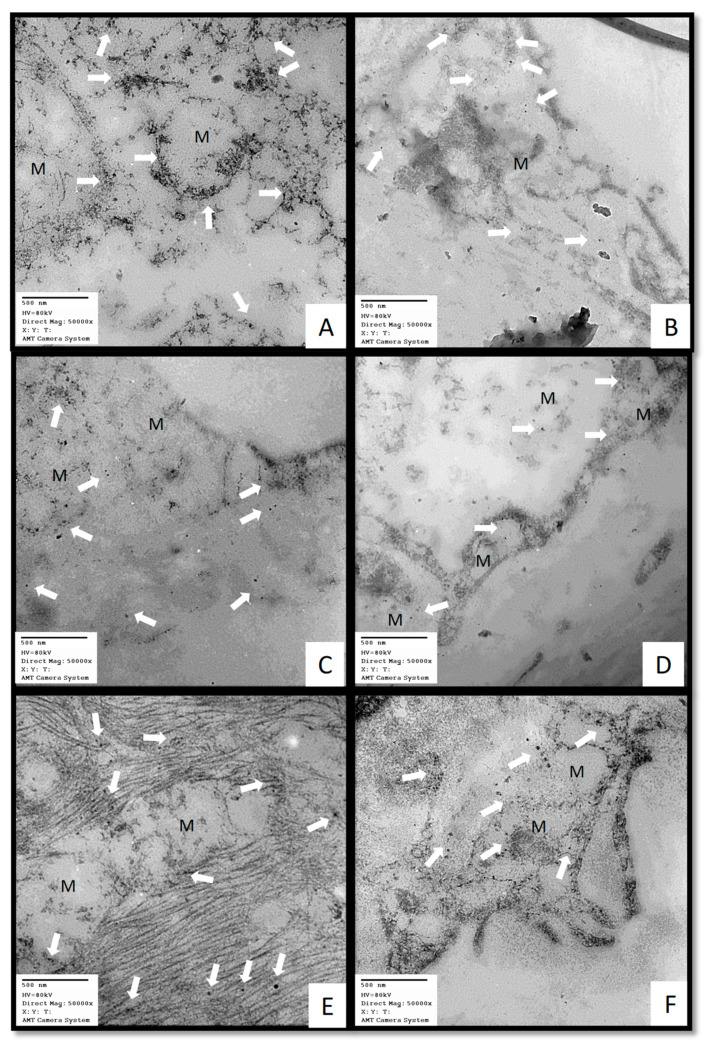
Representative electron micrographs of lung tissue. (**A**) Cyt C, (**C**) COX II, and (**E**) GPx4—from COVID-19 patient; postmortem sample from a 68-year-old female patient with COVID-19 that had associated comorbidities of ischemic heart disease, type II diabetes, morbid obesity, and hypertension. (**B**) Cyt C, (**D**) COX II, and (**F**) GPX4—postmortem sample from a biopsy from a 59-year-old female control subject with hypertension, obesity, type 2 diabetes, and pneumonia. The arrows indicate the presence of the immune colloidal for Cyt C, COX II and GPx4. In patients with COVID-19, rupture of the mitochondrial membrane was evident. The images were taken at 50,000× with a Jeol JEM-1011 electron microscope (JEOL Ltd., Tokyo, Japan) powered to 60 kilovolts and equipped with AMT 542.391 analysis software. Abbreviations: Cyt c = cytochrome C, COX II = cytochrome c oxidase subunit II, and GPx4 = glutathione peroxidase 4. M = mitochondia.

Figure 4 shows the representative electron micrographs of the pneumocytes (type II) of the lung tissue. The results from using the immune colloidal gold technique show that cytochrome C (Cyt c), cytochrome c oxidase subunit II (COX II), and glutathione peroxidase 4 (GPx4) (panels A, C, and E, respectively) were present in the cytosol outside and inside the mitochondrial outer matrix in a COVID-19 patient versus in a postmortem sample from a control subject (panel B, D, and F, respectively), where the marks of the colloidal gold were inside the mitochondria.

## 3. Discussion

In this study, we evaluated whether treatment with MT contributes to decreasing the OS present in COVID-19 patients and whether it may correct the alteration in redox homeostasis. MT reduces the presence of the renin angiotensin receptor in cell membranes, lowering the expression of the ACE2 receptor in the lipid rafts of the cell membrane, which may reduce the entry of the SARS-CoV-2 virus [21]. MT also inhibits the M^pro^, a cysteine protease necessary for SARS-CoV-2 replication [22]. Despite the aforementioned, MT is not virucidal but may indirectly aid in antiviral actions counteracting the alterations that originate from infection in patients. These benefits of MT can be attributed to its properties as an inducer of antioxidant enzymes, stimulator of the immune function, and scavenger of free radicals, and it also may increase the synthesis of GSH [23].

In this sense, our results show that OS markers such as the concentration of total carbonyls and the LPO index decreased in the serum from COVID-19 patients treated with MT, but also that the TAC, NO_2_^−^ and thiol group levels, GSH concentration, and GST activity were increased after the treatment with MT. In infection with SARS-CoV-2, OS is present, and it is characterized by decreases in the GSH tripeptide, thiol group, NO_2_^−^, and GST activity that favor the LPO index and the oxidative background, which leads to diminishing the TAC [24]. In this sense, the ferroptosis present in COVID-19 patients favors LPO when the unsaturated fatty acids are oxidized by the OH^−^. Ferroptosis occurs due to the hijacking of the function of mitochondria, the escape of components of the mitochondrial transport chain into cytosols such as Cyt c and COX II, the knockdown of the GPx4 isoform, and the destruction of the heme group in some enzymes by hypochlorous acid (HOCl) in infection by the SARS-CoV-2 virus [2]. Our results agree with other previously mentioned reports and suggest that treatment with MT may be utilized as an antioxidant adjuvant therapy in fighting SARS-CoV-2 infection since it may decrease the OS in COVID-19 patients. Based on the chemical structure of MT, it may penetrate the lipid membrane bilayer of the cell and protect cell organelles against the damage by ROS caused by different viral infections [25].

With respect to activities of the enzymes that use the GSH such as GPx, GST, GR, and TrxR, which were analyzed in this study, it has been described that infection by the SARS-CoV-2 virus leads to decreases in their activities or expressions [26]. However, treatment with MT may restore the low activities of these enzymes since it increases the GPx, GR, SOD, and catalase gene expressions, and this effect is dose-dependent [27]. In this sense, treatment with MT enhances the mitochondrial SOD expression in elderly rats and prevents the reduction of SOD/GPx and GR/GPx ratios [28]. Another investigation demonstrated that treatment with MT increases the activity of SOD and the levels of GSH and decreases NOx levels in renal ischemia/reperfusion injury in rats [29]. MT can stimulate the ERK1/2 signaling pathway in the presence of excessive ROS through the MT1 and MT2 receptors, and this leads to increases in the expressions of antioxidant enzymes such as GPx, GR, GST, SOD, and GSH via the stimulation of Nrf2 [30].

Our results show that the activity of ecSOD, which is an SOD isoform that has Cu/Zn in the catalytic center, in carrying out the dismutation processes from O_2_^−^ to H_2_O_2_ decreased in the serum of the COVID-19 patients. This was probably due to the excess O_2_^−^ substrate [26] and suggests that this enzyme is incapable of dismutating the O_2_^−^ to H_2_O_2_, resulting in an increase in OS. The activity/expression of this enzyme is reduced in SARS-CoV-2 infection [26,31]. However, the treatment with MT increased the ecSOD activity. This result may seem paradoxical because the dismutation process favors the decrease in O_2_^−^ and an increase in the H_2_O_2_ levels. However, this radical is the substrate of GPx, GST, TxrR, catalase, and peroxidase families and is detoxified to molecular O_2_ and H_2_O. Our results showed that the activities of these enzymes were increased by the treatment with MT. In addition, the infiltration of neutrophils and monocytes that is associated with SARS-CoV-2 infection can activate myeloperoxidase (MPO), which is a peroxidase that participates as a mechanism of defense to decrease the viral infection, through the synthesis and liberation of HOCl. This molecule is a highly potent oxidant agent. However, a high concentration of HOCl can increase the cytokine storm and O_2_^−^ and H_2_O_2_ in the infectious processes [32]. In the early stages of the disease, this mechanism plays a crucial role in neutralizing and destroying viral and bacterial proteins [32,33]. However, as already mentioned, it can exert significant cytotoxic effects when released in large amounts such as in SARS-CoV-2 infection [34]. For example, HOCl can compete with O_2_ at the hemoglobin heme binding sites and cause heme degradation and a subsequent release of Fe^2+^ that triggers the Haber–Weiss and Fenton reactions and contribute to the ferroptosis and LPO present in COVID-19 patients [34]. Also, high concentrations of HOCl can decrease the activities of other enzymes such as lactoperoxidase, eosinophil peroxidase, thyroid peroxidase, and other peroxidase super family members, even myeloperoxidase itself (inhibition of the product of the enzymatic activity) [34,35]. Our results show that the activities of the peroxidases decreased in the serum of the COVID-19 patients and that the treatment with MT favored the activities of these enzymes.

These results suggest that the peroxidase activities are decreased in COVID-19 patients, probably due to the HOCl increase by the severe infection [36]. However, treatment with MT favors the activities of these enzymes and contributes to decreasing the OS [37]. In addition, HOCl overproduction can also mediate the destruction of the heme of the eNOS through a mechanism similar to the destruction of the heme in hemoglobin, which results in a decrease in NO levels [38]. In this sense, the results show that NO_2_^−^ decreased but the treatment with MT restored it. The NO_2_^−^ is a metabolite of the NO, and the hypoxic condition in COVID-19 patients favors a decrease in the O_2_ concentration. Optimal concentrations of O_2_ are necessary for NO synthesis via the eNOS pathway [39]. In ARDS and COPD associated with SARS-CoV-2 infection, the hypoxic conditions contribute to inflammation and HOCl overproduction, which favor the OS and NO decreases [40]. Also, the increase in the formation of O_2_^−^ due to the loss of the activity of ecSOD may react with the scarce NO that has been synthesized and thus contribute to decreasing its concentration, which is associated with the eNOS heme destruction by HOCl. This could contribute to disrupting the redox homeostasis. Furthermore, the scarce NO synthetized might be oxidized by ROS to peroxinitrite, which is more aggressive and may contribute to the inflammatory process associated with the interleukin storm that contributes to the damage in the lungs in COVID-19 patients [41].

Different interleukins participate in the inflammatory processes, including IL-6. Our results show an increase in the prevalence of this interleukin, but treatment with MT decreased it in the serum of the COVID-19 patients. These results suggest the capacity of MT to modulate the inflammatory process. In this sense, a systematic study showed that treatment with MT in a dosage of 5–25 mg/day favored a decrease in the inflammatory markers including IL-6, CRP, and TNF-α in the plasma of COVID-19 patients [42]. In addition, in another clinical study involving 20 hospitalized Iranian patients with mild to moderate COVID-19 severity who received an MT supplement of 9 mg/day for 14 days, there was a significant reduction in TNF-α, IL-1β, IL-2, IL-4, IL-6, and IFN-γ levels via the NLRP3 inflammasome activation, and increased levels of SOD and MT contributed to a decrease in the OS status in comparison with the control group [43]. A meta-analysis that included 13 studies in COVID-19 patients where MT was administered showed that there were significant reductions in TNF-α and IL-6 levels [44]. Therefore, previous studies and our results confirm that administration with MT in COVID-19 patients decreases the pro-inflammatory state and the interleukin storm. These studies also confirm that the doses of MT utilized in our study are within the pharmacological range and that this molecule is effective in fighting SARS-CoV-2 infection. In this sense, a cross-sectional clinical trial with adjuvant therapy found that a dose of 3 to 10 mg of MT shows preventative and therapeutic effects against SARS-CoV-2 infection [45]. Different studies in a mast cell line suggested that MT can exert its anti-inflammatory effects through its receptors [46]. Another study showed that MT may exert anti-inflammatory effects through the regulation of sirtuin-1, which inhibits the differentiation of macrophages towards the pro-inflammatory type [47]. Therefore, MT is a significant inhibitor of the activation of the NLRP3 inflammasome by macrophages [48]. Table 4 shows the previously reported effects of different doses of treatment with MT in patients from both genders with COVID-19 in clinical assays.

On the other hand, MT is capable of modulating glucose homeostasis and energy metabolism, contributing to a decrease in the glucose concentration in the serum of patients with COVID-19 after treatment with MT. In this sense, the administration of complementary MT has demonstrated successful outcomes in the management of diabetes and metabolic syndrome [61] through the re-routing of pyruvate metabolism from the cytosol to the mitochondria. In other words, MT may function as a glycolytic agent since it inhibits the Warburg effect [62]. The Warburg effect is present in SARS-CoV-2 infection, and it is associated with the hijacking of mitochondrial function by the virus to promote its replication [2]. In this condition, it promotes the switching to aerobic glycolysis that leads to the elevation of lactate dehydrogenase and lactic acidosis with Cyt c and COX II liberation that promotes hyperferritinemia [63]. The Warburg state favors the upregulation of the inducible factor 1 alpha (HIF-1α) that contributes to conditions of hypoxia in ARDS [64]. In the mitochondria, Cyt C interacts with H_2_O_2_ and is converted to oxoferryl Cyt c with pseudoperoxidase activity. The oxoferryl derivative of Cyt c oxidizes MT. These result in the restoration of the normal redox cycle of Cyt c, which is essential for the maintenance of mitochondrial bioenergetics that contribute to fighting SARS-CoV-2 infection [65].

In addition to the production of MT by the pineal gland, the mitochondria also synthesize MT, and this production is inhibited by SARS-CoV-2 infection [66]. However, treatment with MT can favor the inhibition of the mitochondrial permeability transition pore and optimize the mitochondrial oxidative phosphorylation that prevents the release of Cyt c and COX II and cardiolipin peroxidation [25]. Furthermore, the circadian gene, Bmal1, which regulates the mitochondrial melatonergic pathway, is inhibited during SARS-CoV-2 infection. Treatment with MT is capable of promoting Bmal1 activity, and this contributes to inhibiting viral replication [24]. In other words, the MT may partly shift glucose metabolism from anaerobic glycolysis to aerobic mitochondrial oxidative phosphorylation and consequently result in decreased lactate production. Lactate is increased in COVID-19 patients [67]. In this sense, our results from using the immune colloidal gold technique show that Cyt c and GPx4 were present in the cytosol, outside and inside the mitochondrial outer matrix of the type II pneumocyte. These results confirm what was previously mentioned about the hijacking of mitochondrial function by the SARS-CoV-2 virus since both Cyt c and GPx4 are mitochondrial and should be inside and not outside the mitochondria [2].

On the other hand, the administration of MT may reduce the use of pain relievers, sedation, agitation, and anxiety, improving the quality of sleep in COVID-19 patients. Furthermore, MT restores the optimal circadian pattern of the sleep/wakefulness cycle, which improves the clinical condition of the person with SARS-CoV-2 pneumonia [36].

**Figure 5:**
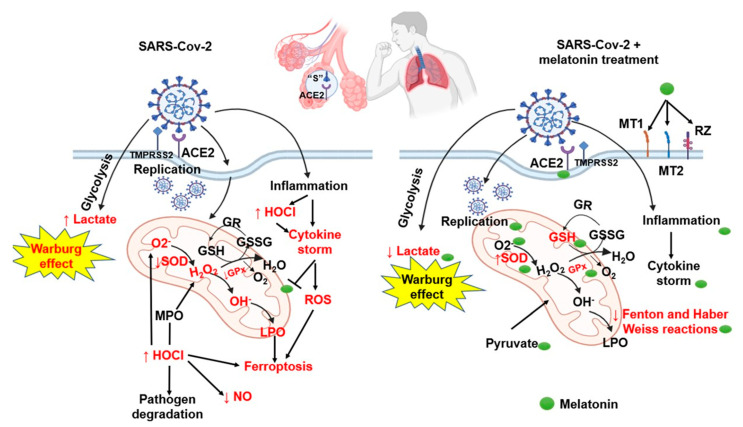
The adjuvant therapy with the treatment with MT may contribute to restoring the redox homeostasis that is altered in COVID-19 patients; favors the activity of the antioxidant enzymes such as GPX, GST, GR, ecSOD, and peroxidases; and increases the levels of markers of OS such as thiols, GSH, and NO_2_^−^. This contributes to decreasing the carbonyl levels and LPO, leading to an increase in the TAC. Furthermore, MT is capable of modulating glucose homeostasis because it functions as a glycolytic agent since it inhibits the Warburg effect. Abbreviations: GPx = glutathione peroxidase, GR = glutathione reductase, GSH = glutathione, GSSG = oxidized glutathione, H_2_O_2_ = hydrogen peroxide, LPO = lipoperoxidation, O_2_^−^ = superoxide anion, OH = hydroxyl, SOD = superoxide dismutase, HOCl = hypochlorous acid, ACE2 = angiotensin-converting enzyme 2, MT = melatonin receptor, and TMPRSS2 = serine protease.

Figure 5 summarizes the possible benefits of treatment with MT in patients with COVID-19.

## 4. Materials and Methods

### 4.1. Population That Comprised the Study

**Figure 6:**
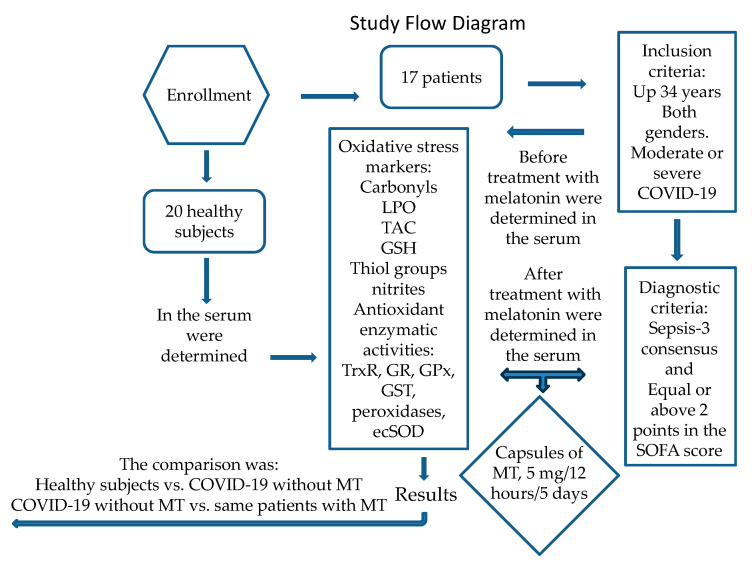
Study flow diagram.

This was a longitudinal (before–after), open, analytical, and prospective study run in 17 patients with COVID-19 who received melatonin (MT) treatment. It compared the results in patients with those from 20 healthy subjects. Inclusion criteria: The COVID-19 patients were 34 years old or more, admitted to the intensive care unit (ICU) of the CITIBANAMEX Center, and they developed (or did not develop) septic shock secondary to moderate or severe pneumonia through SARS-CoV-2 infection. The MT treatment was applied between August and September of 2020. Ethical approval was obtained on 19 August 2020 (Control-9867/2020, register REG. CONBIOETICA-09-CEI-01120160627). The protocol was registered (TRIAL REGISTRATION: ClinicalTrials.gov; Identifier: NCT04570254). Sepsis-3 consent was used for diagnostic criteria of septic shock [68]. An informed consent form written for recruitment and the use of patient data was obtained from each patient or their legal representative, in accordance with the Helsinki Declaration. COVID-19 patients were considered to have septic shock when there was an acute increase of at least 2 points in the Sequential Organ Failure Assessment (SOFA) score [69], which is the scale for assessing the condition of the patients during their stay in the ICU and which includes the stages of neurological, respiratory, hemodynamic, hepatic, and hematologic conditions; had lactate levels ≥ 2 mmol/L; and were dependent on a vasopressor for at least 2 h before recruitment. The SOFA score was evaluated at admission and during the days of the treatment to determine organ dysfunction [70]. Hospitalized patients with COVID-19 were classified as severe or moderate according to their ventilatory status. COVID-19 patients with the severe condition required invasive mechanical intubation according to the Berlin criteria for ARDS [71,72]. Exclusion criteria were as follows: patients that were under chronic use (last 6 months) or recent use of MT, antioxidants, statins, and steroids; patients that were not able to grant informed consent, or refused to be included; and pregnant women or women that were breast feeding. Patients were given individualized management according to an algorithm suggested by Soto et al., and they were not given hydroxychloroquine or antivirals [1]. The patients had not been vaccinated against SARS-CoV-2 because a vaccine had not yet been approved at the time this study was carried out. Some results related to this study were previously reported by Chavarría et al. during the before-and-after-treatment-with-MT treatment evaluation [71]. Figure 6 describes a flow chart of the recruitment and MT treatment in all COVID-19 patients.

### 4.2. Detection of SARS-CoV-2 via Real-Time Reverse Transcriptase Polymerase Chain Reaction

Swab samples were collected from 16 patients infected with SARS-CoV-2 to apply the Paired Technique (nasopharyngeal and saliva). Samples were considered positive for SARS-CoV-2 when both the N1 and N2 protein primer presets were detected. Specific probes to detect the virus and the real-time reverse transcriptase polymerase chain reaction technique (qRT-PCR) were employed to determine the presence of the SARS-CoV-2 virus.

### 4.3. Healthy Subjects

Fifteen men and five women matched by age and gender and negative for SARS-CoV-2 infection were included in the group of healthy subjects. Inflammatory, degenerative, thyroid, and autoimmune diseases; diabetes mellitus; dyslipidemia; and arterial hypertension were not present in the healthy subjects. The intake of antioxidant and non-steroidal anti-inflammatory drugs in healthy subjects that could interfere with the results of the study was suspended 48 h before the samples were obtained. Biochemical variables such as glucose, uric acid, cholesterol, triglyceride, HDL, LDL, C-reactive protein levels and atherogenic index were determined.

### 4.4. Therapeutic Management

During hospitalization, the treatment was chosen according to standard maneuvers, and the requirements of each individual patient, the hemodynamic and electrolyte demands, and the ventilator demands were considered. Treatment was begun before the recognition of the presence or absence of septic shock and during the first hour after admission.

Depending on the hemodynamic status, management with crystalloid solutions and/or albumin was taken into account by means of dynamic indicators. If necessary, vasopressors were used to keep a mean arterial pressure (MAP) ≥ 65 mmHg. Inotropic drugs (dobutamine) were administered when myocardial dysfunction was present. Norepinephrine (NE) was the first option and/or vasopressin was used when there was a need to increase the MAP or reduce the NE dose. When there was a decrease in hemoglobin level (<7.0 g/dL) in the absence of severe hypoxemia, myocardial ischemia, or severe bleeding, transfusion of blood packs was used. Mechanical ventilation with initial volume of 6 mL/kg was used in ARDS patients [69,71,72]. Plateau pressure was maintained at ≤30 cm H_2_O and alveolar conduction pressure of ≤13 cm H_2_O. Positive end expiration pressure titration was managed by the use of the fraction made up of oxygen/positive end expiration pressure (FiO_2_/PEEP). The treatment with anticoagulants was based on the Thatched guidelines [73]. Management with the prone position was necessary in patients with PaO_2_/FiO_2_ of ≤150 mmHg [74].

The standard therapeutic management with dexamethasone 8 mg i.v. every 24 h for 7 days was used in all patients for between 1 and 21 days of the onset of symptoms when not counter-indicated. Pentoxifylline tablets of 400 mg were applied every 12 h via oral route or nasal-enteral tube for 5 days [71]. A counter-indication was when there was a requirement of O_2_ > 3 L, progressive requirement of =2, PaO_2_/FiO_2_ ≤ 250 mmHg, O_2_ use plus bilateral infiltrates in the radiography, O_2_ use plus DHL ≤ 250 U/L or ferritin ≥ 300 or DD ≥ 1000 ng/mL, and CPK ≥ 2 times the upper normal value. The following conditions were not considered as counter-indications or relative counter-indications: glucose > 250 mg/dL with hypoglycemic hypokalemia < 3.3 meq, blood pressure > 155/95 mmHg with antihypertensive treatment, glaucoma, triglyceride volume > 500 mg/dL (start treatment), history of known peptic ulcer or bleeding from recent gastrointestinal tract, untreated or decompensated dementia or psychiatric illness, use of non-potassium sparing diuretics, or use of inhaled B_2_ agonists. The next conditions were monitored at follow-up: pre-prandial capillary glucometer (1–7–13 h) for 10 days, even in fasting patients; MAP per shift; and basal potassium every 72 h.

The dose of the MT therapy was 5 mg (10 prolonged-release capsules), given every 12 h via oral route or nasal-enteral tube for 5 days. The presences of comorbidities or of potential allergies or heart rhythm disorders due to each individual history were considered before the treatment with MT [71]. All data entry was monitored at the coordinating center for patient management, with site visits for source data verification.

### 4.5. Peripheral Blood Samples

Peripheral blood samples were collected through venopuncture. The blood samples were centrifuged for 20 min at 936× *g* and 4 °C. The serum was recovered and stored at −30 °C until use. To determine the levels of acute-phase reactants, urea nitrogen, creatinine, glucose, cholesterol, triglycerides, insulin, HDL, LDL, hemoglobin, leukocytes, lymphocytes, platelets, albumin, D-dimer, fibrinogen, ferritin, C-reactive protein, procalcitonin, and interleukin-6 (IL-6), laboratory tests were conducted for the COVID-19 patients. Data from the patient’s medical history including demographic, illnesses prior to SARS-CoV-2 infection, COVID-19 test result, whether mechanical ventilation was used, and treatment type given were used for the analysis of the results. Additionally, OS markers and antioxidant enzymatic system were determined in serum from the COVID-19 patients and HS. The Homeostasis Model Assessment (HOMA) index for the IR was calculated; HOMA−IR = insulin μU/mL × glucose mM/L/22.5, which is the method used for evaluating the IR and insulin sensitivity from basal insulin and glucose concentrations.

### 4.6. Oxidative Stress Markers

#### 4.6.1. Carbonyl Proteins

Carbonyls were detected spectrophotometrically at 370 nm. A quantity of 100 μL of serum was added to 500 μL of HCl 2.5 M. In parallel, another sample was taken with 500 μL of 2,4-dinitrophenylhydrazine and incubated in the dark at room temperature for one hour. At the end of the incubation period, 500 μL of C_2_HCl_3_O_2_ 20% was added and centrifuged at 15,000× *g* for 5 min. The supernatant was discarded. Two washings were performed, adding 1 mL C_2_H_5_OH/C_4_H_8_O_2_. This was incubated for 10 min and centrifuged at 15,000× *g* for 10 min. Finally, 1 mL of CH_6_CIN_3_ at 6 M in KH_2_PO_4_ at 20 mM and pH 2.3 was added, the mixture was incubated again at 37 °C for 30 min, and the absorbance was read [75]. The values such as the levels of carbonyls (nmol/mL of serum) were expressed.

#### 4.6.2. Evaluation of Total Antioxidant Capacity

Total antioxidant capacity (TAC) was detected spectrophotometrically at 593 nm. A quantity of 100 µL of serum was suspended in 1.5 mL buffer mixture (C_2_H_3_O_2_ at 300 mM and pH 3.6, FeCl_3_·6H_2_O at 20 mM, 2,4,6-tris-2pyridyl-s-triazine at 10 mM, and HCl at 40 mM). These reactants were added in a ratio of 10:1:1 *v*/*v*, respectively, after mixing and were incubated at 37 °C for 15 min in the dark [71]. The values were expressed as levels of carbonyls (nM of trolox/mL of serum).

#### 4.6.3. Determination of the Lipid Peroxidation Marker

The malondialdehyde MDA was read spectrophotometrically at 532 nm. A quantity of 100  μL of serum was used for this determination. CH_3_-OH with BHT at 4% plus KH_2_PO_4_ buffer pH 7.4 was added to serum sample and then incubated at 37 °C for 30 min after 1.5 mL of 2-thiobarbituric acid at 0.8 M was added, then incubated at 90 °C for 1 h. After this, 1 mL KCl was added to 5% plus 4 mL C_4_H_10_O, shaken for 30 s, and centrifuged at 4000 rpm for 2 min. The n-butanol phase was extracted and the absorbance was measured [75]. The values such as the levels of malondialdehyde (MDA/mL of serum) were expressed.

#### 4.6.4. Nitrite Determination

The determination of the concentration of nitrites (NO_2_^−^) in serum, using the technique of Griess, was conducted spectrophotometrically at 540 nm. A quantity of 100 μL of serum, previously deproteinized with NaOH at 0.5 N and ZnSO_4_ at 10%, was mixed and centrifuged at 1789× *g* for 10 min, and the supernatant was incubated with 200 μL of sulfanilamide 1% and 200 μL of N-naphthyl-ethyldiamine 0.1%, and the total volume was adjusted to 1 mL [71]. The values were expressed (nM/mL of serum).

#### 4.6.5. Thiol and Glutathione Levels

Determination of thiol groups was performed spectrophotometrically at 415 nm. A quantity of 50 µL of serum was used according to Erel and Neselioglu’s method [76]. The calibration curve was obtained with solution GSSG 1 mg/1 mL. The values were expressed as μM/mL of serum. Glutathione (GSH) levels were detected spectrophotometrically at 412 nm. A quantity of 100 µL of serum was used according to Ellman’s method. The values were expressed as μM/mL of serum.

#### 4.6.6. Determinations of Antioxidant Enzymes That Use GSH

To evaluate the GR, GST, and GPx activities, 100 µL of serum was used, as previously described in different methods [75]. The samples were incubated and monitored at 340 nm for 6 min at 37 °C. The TrxR activity was determined using 100 µL of serum according to the previously described method [75]. The sample was incubated and monitored at 412 nm for 6 min at 37 °C. The TrxR activity was expressed as TNB nmol/min/mL of the serum, with an extinction coefficient of 13,600 M^−1^ cm^−1^. The GR activity was expressed as μmol of reduced GSSG/min/mL of the serum, with an extinction coefficient of 6220 M^−1^ cm^−1^. The GST activity was expressed as units of GS-TNB mol/min/mL of serum, with an extinction coefficient of 14,150 M^−1^ cm^−1^. The GPx activity was expressed as nmol of NADPH oxidized/min/mL of the serum, with an extinction coefficient of 6220 M^−1^ cm^−1^ at 340 nm for NADPH. The extracellular superoxide dismutase (ecSOD) and peroxidase activities were determined through non-denaturing gel electrophoresis [75]. A total of 25 µL of serum was applied directly in non-denaturing 10% polyacrylamide gels. The electrophoresis was carried out at 120 volts for 4 h. For the ecSOD activity, the gel was incubated with nitro blue tetrazolium at 2.45 mM for 20 min, then incubated with buffer of the KH_2_PO_4_ at 36 mM, EDTA at 28 mM, and riboflavin at 28 Μm pH 7.8 and exposed for 10 min to UV light. Purified SOD from bovine erythrocytes with a specific activity of 112 U/mg of protein (Sigma-Aldrich, St. Louis, MO, USA) was used as positive control for calculating the activity of this enzyme. For the peroxidase activity, the gel was washed with distilled water three times, for 5 min, after being incubated with 3 mg/mL 3,3,5,5-tetramethylbenzidine dissolved in CH_3_-OH/CH_3_COOH/H_2_O (1:1:1 *v*/*v*) with H_2_O_2_ (300 μL) for 10 min. A quantity of 35 μL of horseradish peroxidase was loaded to a final concentration of 178.5 μg as a standard. The activities in the ecSOD and peroxidase gels were analyzed using densitometry with a Kodak Image^®^ 3.5 system.

#### 4.6.7. Interleukin-6 Concentration

IL-6 levels were measured in serum samples through an enzyme-linked immunosorbent assay (ELISA) using a commercial kit according to the manufacturer’s instructions (BioLegend, San Diego, CA, USA), and 50 μL of serum was used for this determination.

#### 4.6.8. Obtainment of the Postmortem Biopsies

Postmortem biopsies were obtained according to the method of Soria-Castro et al. (2021). For the lung biopsy, punctures were performed at the apical and sub-clavicular regions of the right and left lungs [2].

#### 4.6.9. Immune Colloidal Gold Technique

Small tissue samples of the lung from the postmortem COVID-19 patients and from the control subject were processed according to the immune colloidal gold technique [2]. The samples were cut and mounted on carbon/formvar-coated nickel grids. They were then incubated with antiserum for 1 h and with the primary antibodies diluted in a 1:20 ratio: cytochrome C (EPR1327) Rabbit Recombinant monoclonal Ab 133504 (Abcam, Cambridge, UK), glutathione peroxidase 4/GPX4 (E-12) mouse monoclonal sc-166570 (Santa Cruz BioTechnology, Dallas, TX, USA), and COX II (d-5), mouse monoclonal IgM sc-514489 (Santa Cruz BioTechnology), were used. Samples were incubated in a moist chamber at 4 °C overnight. The samples were then washed at 24 °C and incubated with a secondary antibody conjugated with colloidal gold diluted in a 1:20 ratio. The antibodies used were Goat Anti-Rabbit gold 12 nm cat. 111-205-144 (Jackson Immuno Research, West Grove, PA, USA) and rabbit-anti-mouse gold 25 nm cat:25352 (Electron Microscopy Sciences, Hatfield, PA, USA), at 24 °C, evaluated with a JEM-1011 microscope (JEOL Ltd., Tokyo, Japan) at 80 kV, equipped with AMT 542.391 analysis software [2].

### 4.7. Statistical Analysis

Continuous variables of the activities of the antioxidant enzymes were expressed as medians, first quartiles, second quartiles, and interquartile ranges with minimum and maximum ranges. Categorical variables were expressed as frequencies and percentages. Continuous variables were compared, with the Mann–Whitney U rank-sum test followed by the normality test (Shapiro–Wilk), between HS vs. before the MT treatment in the COVID-19 patients and with Kruskall–Wallis test before vs. after the MT treatment in the COVID-19 patients. The sample size was calculated through paired test of two correlated means, specifying the standard error of the differences. The calculation was taken according to the data found in our study of the article by Chavarría et al. [71]. The calculation was based on two forms: the first of these was based on the percentage of cases with elevated lipoperoxidation pretreatment and post-treatment, which ranged between 1.75 before and 1.05 after with an estimated Delta of 0.80 and with both α errors of 0.05 and 0.01 as well as powers of 0.84 and 0.99, respectively. We decided to take the calculation with an alpha error of 0.05 and a power of 84, and we included 17 patients even though only 13 were required. SigmaPlot^®^ version 15 (Systat Software Inc., SanJose, CA 95131, USA, EE.UU, North First Street, Suite 360, Jandel Corporation, San Jose, CA, USA) was used to generate the analysis and graphs. Differences were considered statistically significant when *p* ≤ 0.05.

## 5. Conclusions

The utilized treatment with MT favors the activity of the antioxidant enzymes such as GPX, GST, GR, ecSOD, and peroxidases and increases the levels of markers of OS such as thiols and GSH NO_2_^−^. This contributes to decreasing the levels of carbonyls and LPO, leading to an increase in the TAC. Thus, MT restores the redox homeostasis that is altered in COVID-19 patients. Furthermore, the treatment with MT is capable of modulating glucose homeostasis because it functions as a glycolytic agent since it inhibits the Warburg effect that is present after the hijacking of mitochondrial function by the SARS-CoV-2 virus and is characterized by elevation of lactate dehydrogenase, lactic acidosis, and Cyt c and COX II liberation that promotes hyperferritinemia. Therefore, MT can be used as part of adjuvant therapy in fighting SARS-CoV-2 infection.

### 5.1. Perspectives

Although there are currently different vaccines against SARS-CoV-2 that allow us to reduce the severity of the infection, the administration of MT as an adjuvant therapy can enhance the innate immune response, which may contribute to a better response in the rate of production of specific antibodies and strengthen the compromised redox homeostasis and mitochondrial function caused by the viral infection [24].

### 5.2. Study Limitations

One of the limitations of this study was the small group of COVID-19 patients that were included. Also, the length of time of the administration of the antioxidant therapy could have been longer, but this was not possible due to a lack of budget. However, the preliminary results found in this study are very promising. Another limitation was the lack of a group of postmortem samples with MT treatment with the immune colloidal gold technique to demonstrate that the treatment was able to prevent mitochondrial sequestration.

## Figures and Tables

**Table 1:** Demographic characteristics in the COVID-19 patients at admission.

Variables	Median and Min–Max Range
Age years	62 (34–84)
Body mass index	30 (20–37)
**Comorbidities (%)**	
Diabetes Mellitus	11 (65)
Hypertension	7 (41)
Dyslipidemia	10 (59)
Chronic obstructive pulmonary disease	1 (6)
Normal Weight	6 (35)
Overweight	3 (18)
Obesity	8 (47)
**Gasometry and blood biochemistry median and min–max range**	
PaO_2_ (mmHg)	79 (58–152)
PCO_2_ (mmHg)	31 (27–36)
PaO_2_/FiO_2_ (mmHg)	145 (49–243)
SPO_2_/FiO_2_ (mmHg)	160 (50–280)
Heart Rate bpm	84 (58–106)
Mean arterial pressure (mmHg)	82 (65–99)
Temperature °C	36.5 (35.9–37.2)
Creatinine in serum mg/dL	0.80 (0.60–2.5)
Blood urea nitrogen mg/dL	18.6 (5.8–77.7)
Leukocytes 10^3^/µL	10.1 (5.3–22.6)
Lymphocytes 10^3^/µL	0.66 (0.39–1.29)
Platelets 10^3^/µL	275 (180–576)
Ferritin ng/mL	665.7 (175–2354)
D-dimer ng/dL	655 (27–35,200)
C-reactive protein mg/dL	145 (37–308)
Procalcitonin ng/dL	0.40 (0.06–34.7)
**Score median and Min–Max range**	
SOFA	2 (1–8)
APACHE	5 (4–7)
SAPS	26.5 (13–31)
Days with mechanic ventilation	2 (1–2)
Days in ICU	14.5 (6–20)

Abbreviations: bpm = beats per minute, FiO_2_ = fraction of inspired oxygen, ICU = intensive care unit, PaO_2_ = oxygen at arterial pressure, PCO_2_ = carbon dioxide at partial pressure, SPO_2_ = arterial oxygen saturation, SOFA = Organ Failure Sequential Assessment, APACHE = Acute Physiology and Chronic Health Assessment II, and SAPS = Simplified Acute Physiology II Score.

**Table 2:** Blood biochemical variables and HOMA index values in the COVID-19 patients before and after the treatment with MT.

Variable	Before Q1, Q2, Q3	After Q1, Q2, Q3
Glucose (mg/dL)	157.8, 204.3, 266.6 *	100.8, 139.4, 227.1
Insulin (ng/mL)	0.7, 1.30, 2.0	0.4, 1.3, 2.5
HOMA index	5.8, 9.7, 20.4	4.7, 8.6, 23.2
IL-6 (pg/mL)	16.2, 43.2, 84.1 **	7.8, 7.8, 22.0
HDL (mg/dL)	23.8, 29.0, 42.1 **^†^**	29.3, 35.3, 40.0
LDL (mg/dL)	51.6, 62.5, 80.2	54.1, 86.1, 86.8
Total cholesterol (mg/dL)	23, 141, 162	100, 146, 160
TG (mg/dL)	96, 140, 185	178, 205, 742

Before vs. after the treatment with MT. * *p* = 0.05, ** *p* = 0.02, ^†^ NS (0.09). Abbreviations: IL = Interleukin, HDL = high-density lipoproteins, LDL = low-density lipoproteins, and TG = triglycerides. Wilcoxon paired *t*-test was used. Q1 = first quartile, Q2 = second quartile, and Q3 = third quartile.

**Table 3:** Blood biochemical variables in healthy subjects.

Gender	15 Men, 5 Female
Variable	Q1, Q2, Q3
Age	49.0, 60.0, 63.2
Glucose (mg/dL)	67.7, 100.0, 114.0
Uric acid (mg/dL)	5.0, 6.6, 7.4
Cholesterol (mg/dL)	142.5, 166.5, 191.5
HDL (mg/dL)	33.3, 37.0, 41.2
LDL (mg/dL)	78.7, 93.3, 111.0
TG (mg/dL)	98.1, 113.5, 191.2
C-reactive protein (mg/dL)	1.0, 1.8, 3.6
Atherogenic index	2.0, 2.6, 3.0

Abbreviations: HDL = high-density, lipoproteins, LDL = low-density lipoproteins, TG = triglycerides, Q1 = first quartile, Q2 = second quartile, Q3 = third quartile. Atherogenic index = Log (TG/HDL cholesterol).

**Table 4:** Effect of treatment with MT in patients with COVID-19.

Patients	Gender	Treatment with MT	Variable Determination
1	Female	3 mg/day/2 weeks	Insomnia **↓** [49]
67	43 men, 24 women	21 mg/day/no data	CRP, Platelets **↓** [50]
82	32 men, 50 women	2 mg/day/1 month	Symptoms related to COVID-19 **↓** [51]
58	25 men, 23 women	3 mg/day/1 week	No changes in CRP, lymphocytes, and leukocytes, but oxygen saturation **↓** [52]
82	58 men, 24 women	10 mg/day/2 weeks	General symptoms of COVID-19 **↓** [53]
272	120 men, 152 women	2.6 mg/day/2 weeks	No change in mortality [54]
40	23 men, 17 women	2 mg/day/12 days	Sleep **↑** and delirium ↓ [45]
40	No data	9 mg/day/2 weeks	CRP **↓**, IL-4,-2 **↓** [55]
82	58 men, 24 women	10 mg/day/2 weeks	Thrombosis **↓** [56]
40	26 men, 14 women	6 mg/day/2 weeks	CRP **↓** [57]
24	14 men, 10 women	3 mg/day/2 weeks	CRP **↓**, and breathing improved [58]
82	58 men, 24 women	10 mg/day/2 weeks	CRP **↓**, Ferritin **↓,** and D-Dimer **↓** [59]
100	50 men, 50 women	5 mg/day/1 week	Fewer days without mechanical ventilation [60]

Abbreviations: ↑ = increase, ↓ = decrease, IL = interleukins, and CRP = C-reactive protein.

## Data Availability

The datasets generated and analyzed during the current study are available from the corresponding author on reasonable request.
